# Treatment of posterolateral tibial plateau fractures with a rotational support plate and special pressurizer: technical note and retrospective case series

**DOI:** 10.1186/s13018-021-02544-w

**Published:** 2021-06-23

**Authors:** Yu-Feng Chen, Dong Ren, Lin-Dan Geng, Shuang-Quan Yao, Zhao-Hui Song, Liang Guang, Tian-Ci Wang, Peng-Cheng Wang

**Affiliations:** Orthopaedic Trauma Service Center, Major Laboratory of Orthopaedic Biomechanics in Hebei Province, Third Hospital of Hebei Medical University, Shijiazhuang, Hebei Province China

**Keywords:** Posterolateral tibial plateau, Fracture, Rotational support plate, Special pressurizer, Anterolateral approach

## Abstract

**Background:**

In tibial plateau fractures, the posterolateral segment of the tibia plateau is frequently affected and challenging to treat. Although there are many surgical approaches and fixation methods for the treatment of these fractures, all of these methods have *limitations*. We designed a new rotational support plate (RSP) and a special pressurizer that can fix the fracture directly via the anterolateral approach. This method is advantageous because it leads to little trauma, involves a simple operation, and has a reliable fixation effect. This study details the technique of treating these fractures with the RSP and special pressurizer and provides the outcomes.

**Methods:**

From May 2016 to January 2019, the data of 12 patients with posterolateral tibial plateau fractures treated with the RSP and special pressurizer in our hospital were retrospectively analyzed. Postoperative rehabilitation was advised, knee X-rays were taken at follow-ups, and fracture healing, complications, and knee range of motion were assessed. The Hospital for Special Surgery (HSS) knee score and Knee Injury and Osteoarthritis Outcome Score (KOOS) were used to evaluate knee function at the last follow-up.

**Results:**

The average follow-up time of all patients was 16.5 months (range, 12–25 months). The average bony union time was 3.2 months (range, 3–4.5 months). At the last follow-up, the average knee range of motion was 138° (range, 107–145°). The average HSS score was 91 (range, 64–98). The average KOOS Symptoms score was 90 (range, 75–96). The average KOOS Pain score was 91 (range, 72–97). The average KOOS ADL score was 91 (range, 74–97). The average KOOS sport/recreation score was 83 (range, 70–90). The average KOOS QOL score was 88 (range, 69–93). Skin necrosis, incision infections, and fixation failure did not occur during the follow-up period.

**Conclusions:**

With our newly designed RSP and special pressurizer, posterolateral tibial plateau fractures can be easily and effectively reduced and fixed through the anterolateral approach, which serves as a novel treatment for posterolateral tibial plateau fractures.

**Supplementary Information:**

The online version contains supplementary material available at 10.1186/s13018-021-02544-w.

## Background

In tibial plateau fractures, the posterolateral segment of the tibia plateau is frequently affected and challenging to treat [1]. These fractures are often combined with fractures of other parts of the tibial plateau, accounting for 14.3–44.2% of all tibial plateau fractures [1, 2]. The posterolateral tibial plateau is essential for stabilizing the posterolateral knee joint, especially when the knee joint is flexed. If good reduction and fixation are not observed after a fracture, significant discomfort and dysfunction occur.

With the traditional treatment method, the posterior or posterolateral approach is usually used in the knee joint to directly separate and expose the posterolateral platform, reposition the structures under direct vision and place the internal fixator behind the tibia for fixation [3, 4]. Because there are important neurovascular branches in the surgical area (such as the common peroneal nerve), careful anatomical separation is required. In a relatively simple anatomical structure, the anterolateral approach can expose only part of the lateral plateau due to the obstruction of the fibula, so it is difficult to expose the fracture. The fracture needs to be fixed through the supra fibula space, and the fixation effect is often not ideal [5]. Although fibula osteotomy provides more exposure of the posterolateral plateau, fracture healing at the osteotomy site and the stability of the lateral structure of the knee joint are not ideal [6]. Although there are many surgical approaches and internal fixation methods for this type of fracture, methods that have the advantages of simple surgical approaches lead to small intraoperative trauma and have reliable internal fixation effects have not been reported.

**Fig. 1:**
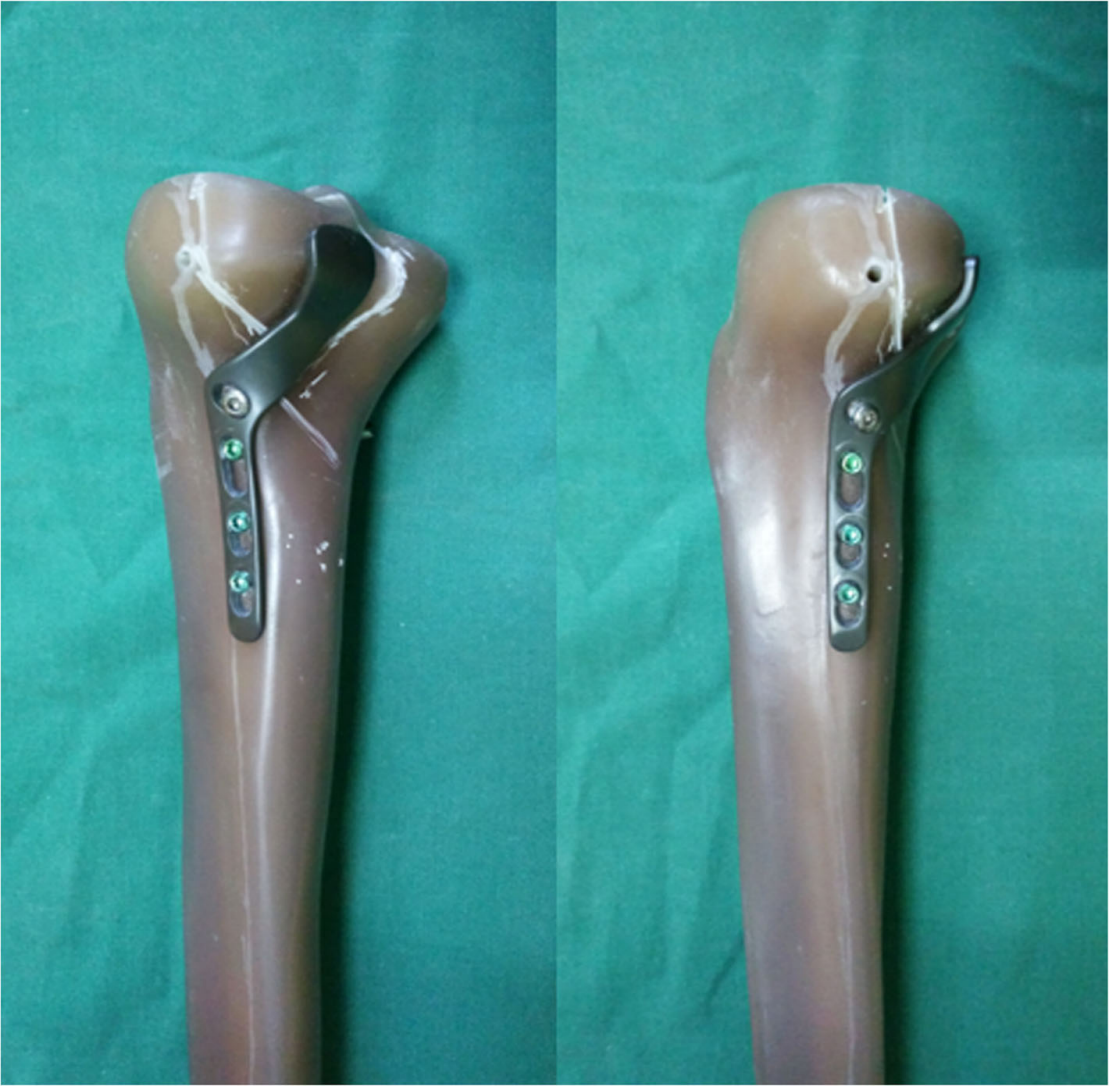
The rotational support plate

**Fig. 2:**
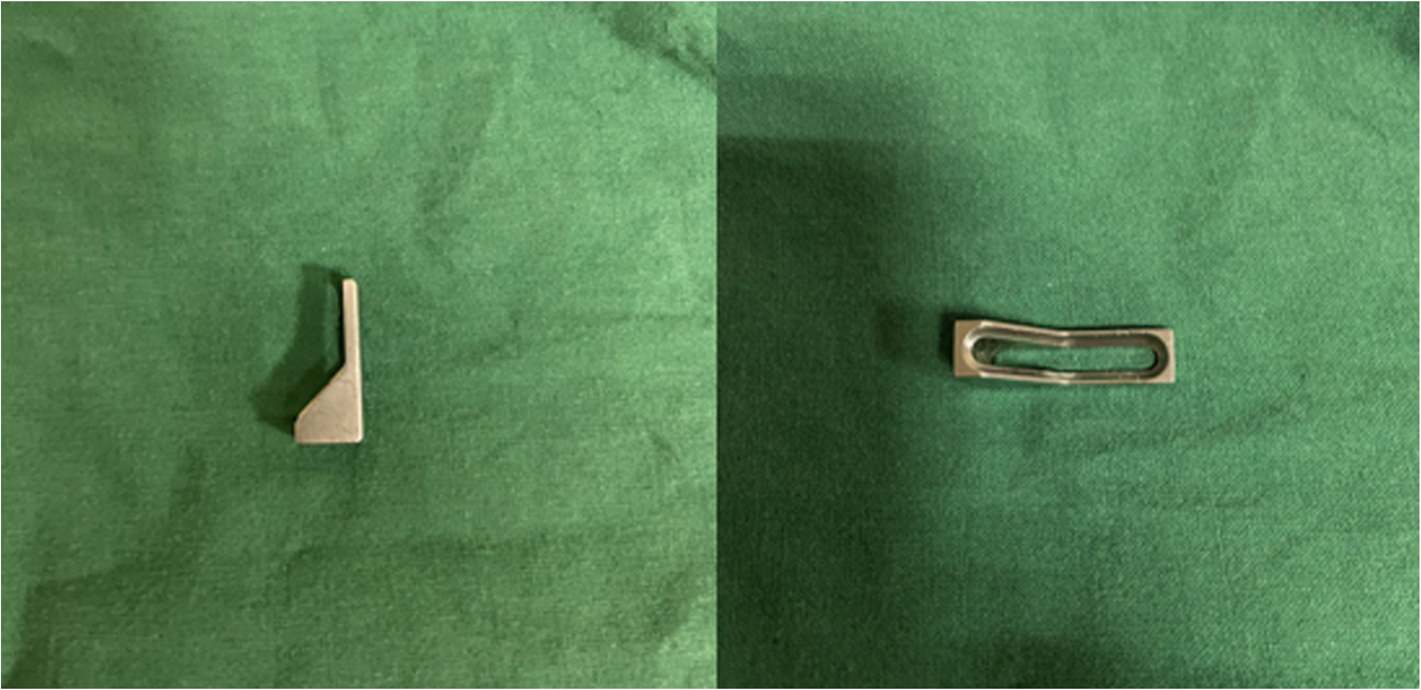
The special pressurizer

To that end, we designed a new rotational support plate (RSP) (Fig. 1) [7]. In previous work, we verified that the RSP has reliable fixation strength by performing biomechanical tests and finite element analysis [8]. In addition, to achieve better compression and reduction effects, we designed a special pressurizer (Fig. 2) for the RSP. Their combined application solves the problems related to the abovementioned surgical approach and fixation effect. Compared with manually pushing the RSP for compression, the special pressurizer is easier to operate, and the compression and reduction effect of the pressurizer is more reliable.

This study includes a clinical case series and presents the method of treating posterolateral tibial plateau fractures with the RSP and special pressurizer.

## Materials

This was a retrospective case series in a single referral hospital. The study was approved by our institutional ethics committee (approval# 2016-005-1). The study group included 12 patients (8 men and 4 women) who had been treated for posterolateral tibial plateau fractures between 2016 and 2019. The average age at the time of trauma was 44.7 years (range, 31–66 years). The mechanism of injury was a motor vehicle accident in three patients and a fall from a height in nine patients. According to the Schatzker classification system, there were 2 cases of type I, 5 cases of type II, 2 cases of type III, 1 case of type IV, and 2 cases of type V fractures. The preoperative imaging examinations included X-ray and CT scans and three-dimensional reconstruction. All operations were performed by the same experienced trauma surgeons after the soft tissue condition stabilized.

## Methods

### Approach

Under general anesthesia or spinal anesthesia, the patient was placed in the supine position, the affected limb was bent at the hip and knee by approximately 30–40°, and the calf was placed in neutral rotation. All RSP fixation procedures were performed using the traditional anterolateral approach.

**Fig. 3:**
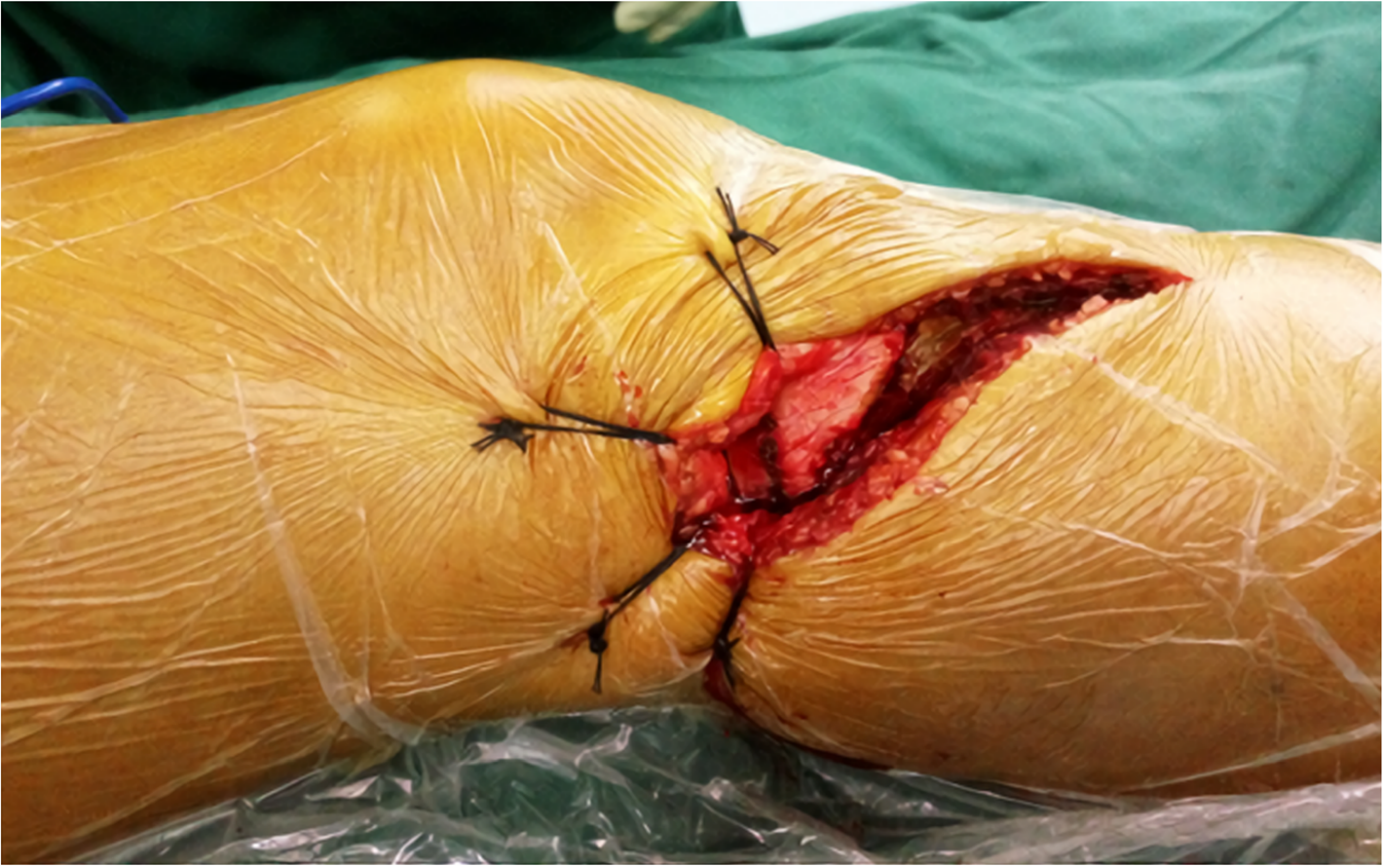
Anterolateral approach

The incision we made started from the lateral joint line of the knee joint, arced forward and downward over the Gerdy tubercle, and extended to the distal end. The subcutaneous tissue was separated, and the iliotibial band was incised subperiosteally along the outer edge of the tibia. The lateral joint capsule and the ligament between the meniscus and tibial plateau were incised. The meniscus was carefully dissected to expose the upper surface of the lateral tibial plateau, while its anterior and posterior attachment were preserved. The lateral meniscus, iliotibial band and superficial tissue were retracted proximally using sutures. The lateral collateral ligament, posterior iliotibial band, popliteal tendon, and superficial tissue were posteriorly retracted using sutures (Fig. 3). After a Schanz nail was placed in the middle and lower sections of the femur and tibia and with slight internal rotation and varus of the lower leg while a femoral distractor was applied, the posterolateral tibial plateau articular surface was fully visualized.

### Reduction and plate fixation

**Fig. 4:**
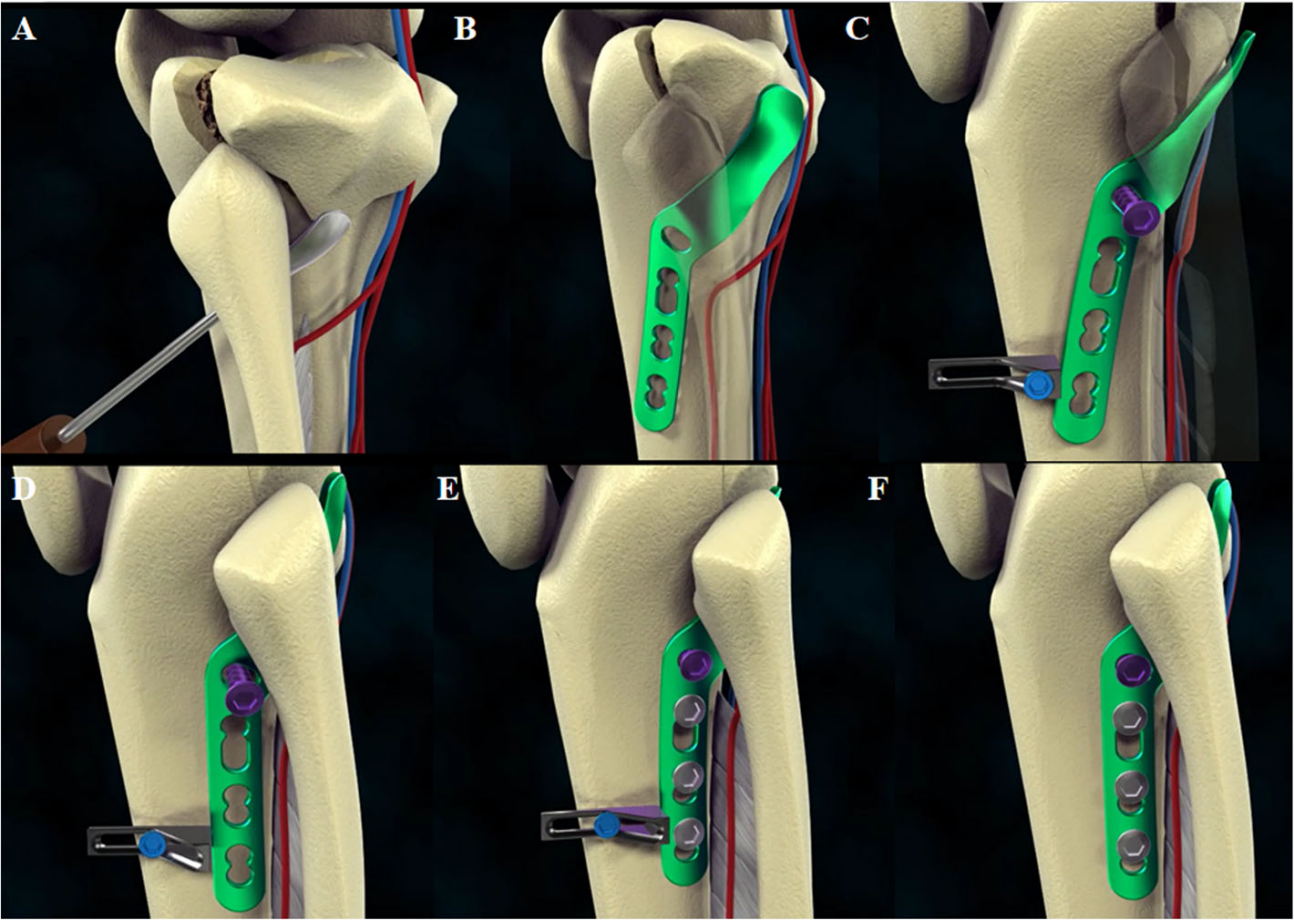
Surgical procedure diagrams. **A** Minitype elevator is used to lift the fracture block of the posterolateral tibial plateau. **B** Placement of the RSP. **C** The special pressurizer is placed close to the distal end of the RSP. **D** By screwing in the screw, the pressurizer is squeezed, thereby pushing the distal end of the RSP so that the fracture can be further compressed and reduced. **E**, **F** Two or three consecutive locking screws are inserted in the distal screw holes, and then the pressurizer is removed

Through the oval hiatus of the interosseous membrane, a minitype elevator was used to peel back the periosteum to the posterior lateral aspect of the tibial plateau and lift the fracture block of the posterolateral tibial plateau (Fig. 4A). The minitype elevator should be placed carefully so as not to damage posterior neurovascular structures. This procedure led to initial reduction of the fracture and established a soft tissue path, which needed to be close to the periosteum to avoid injury to nearby neurovascular bundles, for the implantation of the RSP.

The straight part of the RSP was placed beneath the proximal tibiofibular joint anterolaterally, and the curved part was immediately adjacent to the posterolateral fragments (Fig. 4B). First, a screw was inserted through the sliding hole located at the junction of the straight and curved parts as a fulcrum. For patients with osteoporosis, we recommend bicortical screw placement to ensure reliable support. Then, the special pressurizer was placed close to the distal end of the straight part (Fig. 4C). At this time, by screwing in the screw, the L-shaped hollow frame of the pressurizer was squeezed, thereby pushing the distal end of the RSP so that the posterior lateral fracture could be further compressed and reduced (Fig. 4D). Finally, two or three consecutive locking screws were inserted in the distal screw holes before the pressurizer was removed (Fig. 4E, F). All reduction and fixation procedures were conducted under intraoperative fluoroscopic guidance. The ligaments and joint capsule were carefully sutured back to the attachment, and the subcutaneous tissue and skin were closed over suction drains.

For the patients with anterolateral tibial plateau fractures, the anterolateral tibial plateau was routinely reduced and then used as a reference to reduce the posterior lateral fracture; after reduction, the 3.5-mm lateral anatomic locking plate was used to fix the lateral fracture. For the patients with medial tibial plateau fractures, anteromedial incisions were made, and a 3.5-mm “T”-shaped locking plate or lag screws were used for fixation. Additionally, performing the above internal fixation technique did not affect the placement of the RSP.

### Evaluation index

A CT examination of the knee joint was performed after the operation to observe the state of fracture reduction and fixation. The knee X-rays were taken at 6, 12, 24, and 48 weeks after surgery to observe the level of bony union. The knee joint range of motion was recorded at the last follow-up. Knee joint function was evaluated using the American Hospital for Special Surgery (HSS) knee joint function assessment tool and Knee Injury and Osteoarthritis Outcome Score (KOOS) [9, 10]. The highest HSS score possible is 100 points; scores of > 85 points are excellent, those of 70–84 are good, those of 60–69 are fair, and those of ≤ 59 are poor. The KOOS questionnaire covers five dimensions that are reported separately: pain, symptoms, activities of daily living (ADL), sport and function (sport/recreation), and knee-related quality of life (QoL). Standardized answer options are provided and each question is rated on a scale from 0 to 4. A normalized score (100 indicating no symptoms and 0 indicating extreme symptoms) is then calculated for each subscale. Scores are categorized as “Excellent” (95–100), “Good” (84–94), “Fair” (65–83), and “Poor” (< 64).

## Results

All 12 patients underwent fixation with the RSP through the anterolateral approach. Among them, 10 patients with anterolateral tibial plateau fractures underwent additional fixation with lateral “L”-shaped anatomical locking bone plates, and the remaining 2 patients with medial tibial plateau fractures were additionally treated with the anteromedial approach to expose and reduce the fracture. One case was fixed with a 3.5-mm “T”-shaped locking bone plate, and the other was fixed with an anterior medial screw. All cases had sutures for 14 days after the operation, and the incision healed within the first stage. All 12 patients were followed-up for 1 year, with an average follow-up period of 16.5 months (range, 12–25 months). During the follow-up period, there were no cases of skin necrosis, infection, internal fixation loosening, fracture reduction loss, or other complications.

Bony union was achieved in all 12 cases. The average bony union time was 3.2 months (range, 3–4.5 months). At the 6-week postoperative re-examination, the X-ray films showed that the fractures were in good positions in all cases, and there were no cases of intrasegmental gaps, steps, and loose internal fixation. The patients were then instructed to start walking with partial weight. At the 12-week postoperative re-examination of all cases, 9 cases exhibited bone union. There were no cases of reduction loss after beginning of weight bearing. Two patients exhibited bony union at 14 weeks postoperatively, and 1 patient exhibited bony union at 18 weeks postoperatively. One patient reported pain after walking and has limited range of motion (0–107°). Radiologically, signs of early onset of osteoarthritis were observed.

At the last follow-up, the average knee range of motion was 138° (range, 107–145°). The average HSS score was 91 (range 64–98), and the score was considered excellent in 10 cases, good in 1 case, and fair in 1 case. The average KOOS Symptoms score was 90 (range, 75–96). The average KOOS Pain score was 91 (range, 72–97). The average KOOS ADL score was 91 (range, 74–97). The average KOOS Sport/recreation score was 83 (range, 70–90). The average KOOS QOL score was 88 (range, 69–93). The reason for the one low HSS and KOOS score may have been that after receiving instructions for functional recovery exercises, the patient did not perform knee joint exercises as directed because of fear of refracture.

## Case presentation

**Fig. 5:**
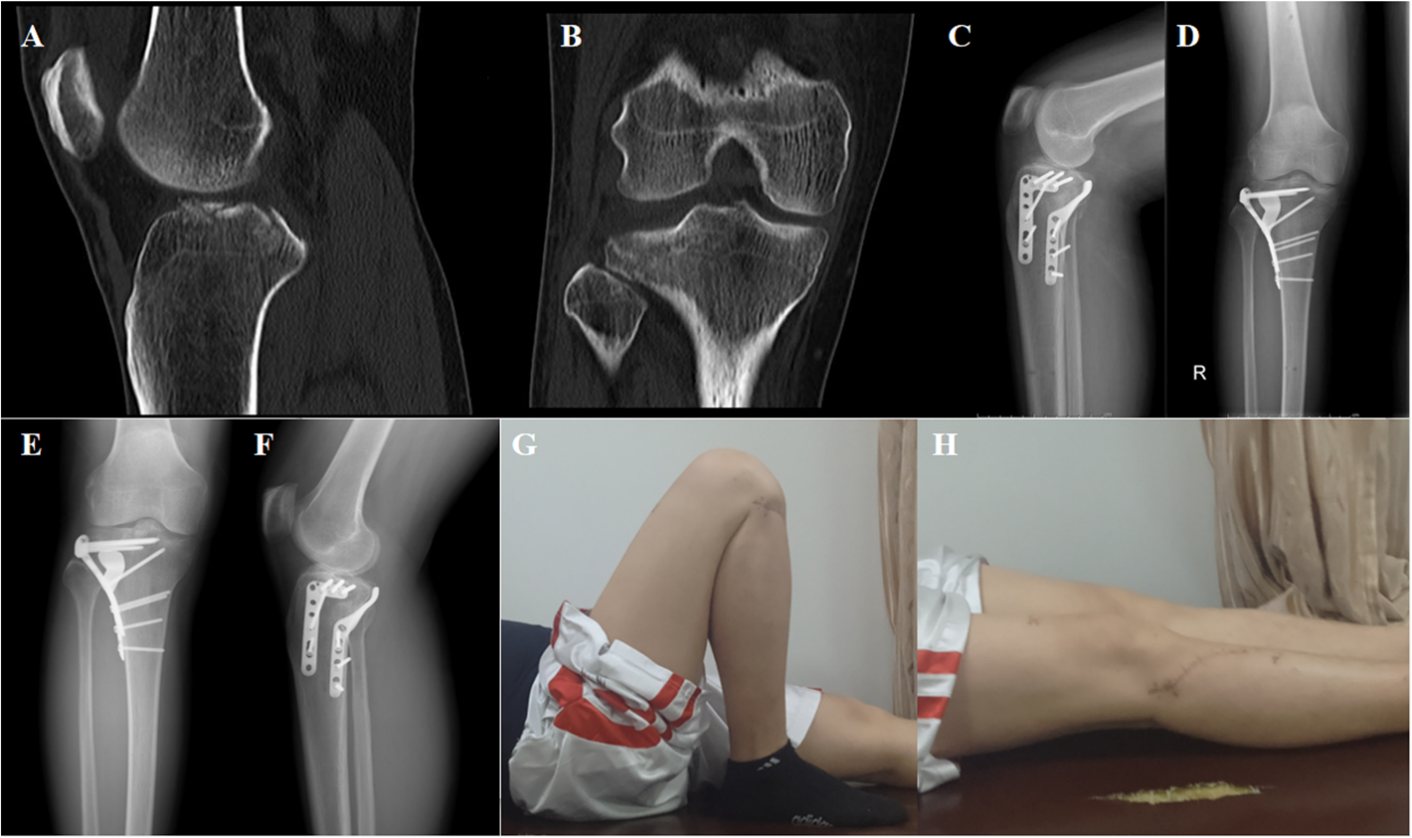
A 39-year-old man who sustained a right posterolateral tibial plateau fracture after a falling injury (Schatzker type II). **A**, **B** Preoperative computed tomography (CT), showing the posterolateral tibial plateau fracture. **C**, **D** Postoperative radiographs, showing the RSP fixation. **E**, **F** Follow-up radiographs after 1 year, showing complete union of fracture. **G**, **H** The clinical outcome was excellent

A 39-year-old man was admitted to our hospital for treatment 10 h after a falling injury. The physical examination revealed right knee joint tenderness with limited mobility. The plain X-ray and CT images showed a right posterolateral tibial plateau fracture (Schatzker type II) (Fig. 5A, B).

Surgical treatment involving open reduction and internal fixation with the RSP was performed. Antibiotics were routinely used to prevent infection 24 h after surgery. The stitches were removed 2 weeks after surgery. X-rays were taken for review at the end of the surgery and at 6 weeks, 3 months, 6 months, 12 months, and 24 months postoperatively (Fig. 5C–F). At the last follow-up, the postoperative result in terms of the HSS score was 98. The final knee joint arc of flexion motion was 135° (Fig. 5G, H). During the 2-year follow-up period, there were no cases of fracture nonunion, necrosis of the femoral head, or other complications.

## Discussion

Posterolateral tibial plateau fractures have always been difficult to treat in clinical practice due to the deep location of the fracture fragment and the complicated surrounding anatomical structure. For this type of fracture, the goal of surgery is to anatomically reduce the articular surface and provide strong fixation while incurring as little intraoperative damage as possible, thereby allowing early functional recovery and reducing the risk of postoperative complications [11, 12]. The technique involving both the RSP and special pressurizer to treat posterolateral tibial plateau fractures leads to little trauma and is a simple operation. It has achieved good curative effects in initial clinical applications.

The RSP was designed based on the anatomical characteristics of the posterolateral tibial plateau. It consists of a straight body, a curved neck, and a slightly wider head. The body is placed on the outside of the tibia. The neck can pass through the interosseous membrane hole, and the head can hold the posterior fracture like a “palm” [7]. The fixation strength of the RSP has been shown to meet the clinical needs through biomechanical and finite element tests [8]. Combined with the special pressurizer, it can simply and effectively allow compression fixation of the posterolateral tibial plateau fracture. Compared with manually pushing the RSP for compression, the method involving the special pressurizer has the following advantages: (1) The compression force can be controlled by adjusting the number of times the screw is turned so that the appropriate compression degree can be selected according to the fracture condition. (2) The pressurizer can generate much higher pressures than can the manual method, and the pressures can be maintained. (3) The simple operation can be completed by a single person, which improves the efficiency of the operation and reduces the number of times fluoroscopy needs to be performed. This technique is suitable for all types of posterolateral tibial plateau fractures, especially for small fracture fragments and comminuted fractures, as it has a good fixation effect.

At present, the clinical surgical approaches for posterolateral tibial plateau fractures include the posterior median, posterolateral, and anterolateral approaches. There are many options, but all of these options have different limitations. Posterior approaches are more often recommended to address the posterolateral tibial plateau segments [1]. The traditional posterior median “S” approach and the inverted “L” approach can directly expose the posterolateral tibial plateau, and reduction and internal fixation can be performed under direct vision [13, 14]. Because there are important neurovascular branches in the surgical area (such as the common peroneal nerve), careful anatomical separation is required. If the posterior plate is long and placed under the blood vessel, there may be a risk of vascular injury. It is often necessary to cut off the medial head of the gastrocnemius muscle to achieve full exposure and fixation, and the severity of intraoperative injury is high. Both of the posterolateral approaches reported by Gavaskar et al. [15] and Pires et al. [6] require dissociation of the common peroneal nerve and fibular osteotomy. Fibula osteotomy can cause iatrogenic fibula fractures, and there may be a risk of postoperative fracture nonunion due to longitudinal traction of the lateral collateral ligament and biceps femoris tendon. Although the modified posterolateral approach proposed by Frosch et al. [16] avoids fibular osteotomy, it still requires separation of the common peroneal nerve, which also increases the risk of nerve damage. The anterolateral approach is blocked by the fibula, which makes it difficult to expose posterolateral plateau fractures. Kfuri et al. [17] reported that the modified anterolateral approach for the treatment of posterolateral tibial plateau fractures increases the level of exposure, but the support and fixation effects of the existing internal fixation devices are limited. Especially for posterolateral comminuted fractures, it is difficult to achieve effective fixation.

The use of the RSP to treat posterolateral tibial plateau fractures through the anterolateral approach can effectively avoid the above problems. The anatomical structure of the anterolateral approach is simple, and the level of intraoperative injury is small. The RSP can be implanted through the space of the upper tibiofibular joint without damaging the important structures around the knee joint. Combined with the special pressurizer, the posterolateral fracture can be effectively compressed and fixed without the need for dissociation of the common peroneal nerve or osteotomy. In addition, the operation is performed with the patient in the supine position, which is convenient for intraoperative operation, and the placement of the RSP does not affect the treatment of concurrent medial, lateral, or anterolateral fractures.

Due to inter-individual anatomical differences, the RSP will not perfectly match everyone’s posterolateral tibial plateau. However, even when the plate cannot be in full contact with the bone surface, it can provide sufficient pressure with the assistance of a special pressurizer, and it will not affect the patient’s recovery. In addition, the number of cases followed-up and the length of the follow-up period in this study were limited, and longer-term follow-up studies with larger sample sizes are still needed to further verify the clinical efficacy of this method.

## Conclusion

With our newly designed RSP and special pressurizer, the posterolateral tibial plateau fractures can be easily and effectively reduced and fixed through anterolateral approach, which provides a novel method for the treatment of posterolateral tibial plateau fractures.

## Supplementary Information

**Additional file 1:.** Surgery animation

## Data Availability

The data of the present study is available from the corresponding author on request.

## Abbreviations

RSP: Rotational support plate
CT: Computed tomography
HSS: American Hospital for Special Surgery

## References

[1] Krause M, Preiss A, Müller G, Madert J, Fehske K, Neumann MV, Domnick C, Raschke M, Südkamp N, Frosch KH (2016). Intra-articular tibial plateau fracture characteristics according to the “Ten segment classification”. Injury..

[2] Sohn HS, Yoon YC, Cho JW, Cho WT, Oh CW, Oh JK (2015). Incidence and fracture morphology of posterolateral fragments in lateral and bicondylar tibial plateau fractures. J Orthop Trauma..

[3] Pierrie SN, Harmer LS, Karunakar MA, Angerame MR, Andrews EB, Sample KM, Hsu JR (2016). Limited added value of the posterolateral approach. J Knee Surg..

[4] Kottmeier SA, Watson JT, Row E, Jones CB (2016). Staged fixation of tibial plateau fractures: strategies for the posterior approach. J Knee Surg..

[5] Krause M, Müller G, Frosch KH (2018). Chirurgische Zugänge bei Tibiakopffrakturen [Surgical approaches to tibial plateau fractures]. Unfallchirurg..

[6] Pires RES, Giordano V, Wajnsztejn A, Oliveira Santana E, Pesantez R, Lee MA, de Andrade MAP (2016). Complications and outcomes of the transfibular approach for posterolateral fractures of the tibial plateau. Injury..

[7] Ren D, Liu Y, Zhou B, Lu J, Wang P. A novel design of a plate for posterolateral tibial plateau fractures based on computed tomography mapping of the proximal tibiofibular joint. Med Sci Monit. 2018;24:9300-9306. Published 2018 Dec 21. 10.12659/MSM.911738 PMC6320643 30574954

[8] Ren D, Liu Y, Lu J, Xu R, Wang P (2018). A novel design of a plate for posterolateral tibial plateau fractures through traditional anterolateral approach. Sci Rep..

[9] Insall JN, Ranawat CS, Aglietti P, Shine J (1976). A comparison of four models of total knee-replacement prostheses. J Bone Joint Surg Am..

[10] Roos EM, Roos HP, Ekdahl C, Lohmander LS (1998). Knee injury and Osteoarthritis Outcome Score (KOOS)--validation of a Swedish version. Scand J Med Sci Sports..

[11] Liang J, Zhang Q, Liu P, Wang B, Zhou X, Chen G, Zhang C, Xu Y (2018). Arthroscopic-assisted inflatable bone tamp reduction for treatment of posterolateral tibial plateau fractures. Injury..

[12] Özdemir G, Yilmaz B, Şirin E, Keskinöz EN, Kırıkçı G, Bayramoğlu A (2018). The anatomical relationship of the neurovascular structures in direct posterior lateral gastrocnemius split approach for posterolateral tibial plateau fractures. Eur J Trauma Emerg Surg..

[13] Connolly JF (2005). The posterior shearing tibial plateau fracture: treatment and results via a posterior approach. J Orthop Trauma..

[14] Lin KC, Tarng YW, Lin GY, Yang SW, Hsu CJ, Renn JH (2015). Prone and direct posterior approach for management of posterior column tibial plateau fractures. Orthop Traumatol Surg Res..

[15] Gavaskar AS, Gopalan H, Tummala NC, Srinivasan P (2016). The extended posterolateral approach for split depression lateral tibial plateau fractures extending into the posterior column: 2 years follow up results of a prospective study. Injury..

[16] Frosch KH, Balcarek P, Walde T, Stürmer KM (2010). A new posterolateral approach without fibula osteotomy for the treatment of tibial plateau fractures. J Orthop Trauma..

[17] Kfuri M, Schatzker J, Castiglia MT, Giordano V, Fogagnolo F, Stannard JP (2017). Extended anterolateral approach for complex lateral tibial plateau fractures. J Knee Surg..

